# Identification of Calcium-Sensing Receptor (CAS) as a Phytaspase Interactor: Implications for the CAS Trafficking Pathway

**DOI:** 10.3390/ijms27209202

**Published:** 2026-10-16

**Authors:** Anastasia V. Litvinova, Artemii A. Pigidanov, Tatevik A. Torosian, Victor G. Zgoda, Marina V. Serebryakova, Irina E. Kovaleva, Mahir D. Mamedov, Sergei A. Golyshev, Grigoriy G. Safronov, Nina V. Chichkova, Andrey B. Vartapetian

**Affiliations:** 1 Belozersky Institute of Physico-Chemical Biology, Lomonosov Moscow State University, Moscow 119991, Russia; nasta.litwinova@gmail.com (A.V.L.); artemiy.pigidanov@mail.ru (A.A.P.); mserebr@mail.ru (M.V.S.); kovaleva@belozersky.msu.ru (I.E.K.); mamedov@belozersky.msu.ru (M.D.M.); grigorij.safronov@gmail.com (G.G.S.); chic@belozersky.msu.ru (N.V.C.); 2 Faculty of Bioengineering and Bioinformatics, Lomonosov Moscow State University, Moscow 119991, Russia; tatoshik312@gmail.com; 3 Institute of Biomedical Chemistry, Moscow 119121, Russia; victor.zgoda@gmail.com

**Keywords:** calcium-sensing receptor, chloroplast, phytaspase, plant cell death, plasma membrane, protein trafficking, proteolysis

## Abstract

Proteolytic enzymes play important and diverse roles in regulating protein homeostasis in living cells. Phytaspase, a member of the plant subtilisin-like protease family possessing a ‘caspase-like’ selectivity and specificity of hydrolysis of its targets, is a cell death-related protease. Phytaspase displays a peculiar trafficking pattern and is involved in the execution of plant cell death induced by biotic and abiotic stresses. To elucidate the molecular mechanisms of phytaspase functioning, a search for *Nicotiana tabacum* phytaspase (*Nt*Phyt)-interacting proteins was performed using the BioID approach. This search identified the calcium-sensing receptor (CAS) protein as a candidate *Nt*Phyt interactor. Direct interaction of *Nt*Phyt with *N. tabacum* CAS (*Nt*CAS) was corroborated by an in vitro analysis utilizing the purified proteins. Furthermore, *Nt*Phyt was shown to introduce five cleavages in the CAS molecule in vitro. For *Nt*CAS-EGFP produced in *N. benthamiana* leaves, dual localization to chloroplasts and the cell periphery, including the plasma membrane, was observed, whereas *Nt*Phyt proteolytic activity was undetectable in the chloroplasts. A CAS precursor protein apparently preserving the chloroplast transit peptide was found to accumulate upon a light-to-dark transition and to disappear in the presence of elevated levels of *Nt*Phyt. A requirement for CAS for stress-induced retrograde transport of *Nt*Phyt was uncovered, and partial co-localization of CAS with *Nt*Phyt was observed in cells subjected to oxidative stress. Our results are consistent with the model according to which CAS transiently localizes to the plasma membrane as an intermediate location on its way to its final destination, the chloroplasts, and interacts with *Nt*Phyt at this stage.

## 1. Introduction

In living cells, the behavior of proteins is somewhat reminiscent of that of humans. Some proteins are typical homebodies, while others travel long distances to reach their destinations and are ready to change their location depending on the external cues [1,2,3,4,5,6]. Phytaspases, a cell death-related group of plant subtilisin-like proteases (subtilase family), definitely belong to the second group. Phytaspases possess an exceptional selectivity and ‘caspase-like’ aspartate specificity of hydrolysis of their protein targets (hence, their name) [7,8,9,10]. Being synthesized as inactive preproenzymes, phytaspases, like most plant subtilases, pass through the endoplasmic reticulum (ER) and Golgi apparatus on their way to the apoplast. In the course of this anterograde journey, phytaspase precursors become constitutively and autocatalytically processed (and thus activated) within the plant cell, and the mature enzyme is eventually released into the apoplast [7]. However, upon perception of biotic and abiotic death-inducing insults, phytaspases utilize clathrin-mediated endocytosis (CME) to re-enter the plant cell and gain access to their intracellular targets [7,11,12]. A mechanism allowing these water-soluble extracellular enzymes to employ CME for stress-induced ‘retrograde transport’ is not well understood, which prompted a search for phytaspase-interacting proteins that might contribute to the stress-induced internalization of phytaspases. For *Nicotiana tabacum* phytaspase (*Nt*Phyt), two such partners were identified previously, the soluble ER chaperone calreticulin 3 [13] and the intracellular Tubic protein (*N. tabacum* Tubby-like F-box protein 8) associated with phospholipids of the plasma membrane [14]. In continuation of this search, we used a combination of an in vivo BioID (proximity-dependent biotin identification) approach and in vitro analysis utilizing the purified proteins to identify *N. tabacum* calcium-sensing receptor (*Nt*CAS) as a *Nt*Phyt partner and target.

*Arabidopsis thaliana* CAS was initially identified as a protein mediating the increase in the concentration of cytosolic Ca^2+^ ([Ca^2+^]_cyt_) in response to an increase in extracellular Ca^2+^ concentration ([Ca^2+^]_ext_) [15]. CAS is expressed predominantly in shoots, including guard cells, and down-regulation of CAS using CAS antisense constructs disrupted [Ca^2+^]_ext_ signaling in guard cells. The CAS antisense plants showed a severe defect in bolting in response to Ca^2+^ deficiency [15]. CAS was also demonstrated to synchronize diurnal [Ca^2+^]_cyt_ oscillations in plants with [Ca^2+^]_ext_, the reduced [Ca^2+^]_cyt_ oscillations in CAS antisense plants being attributed to impaired [Ca^2+^]_ext_ sensing [16]. At the whole-leaf level, CAS antisense plants were impaired in [Ca^2+^]_ext_-induced inositol 1,4,5-triphosphate (IP_3_) production, thus potentially coupling CAS functioning with the release of Ca^2+^ from internal stores [16].

**Figure 1:**
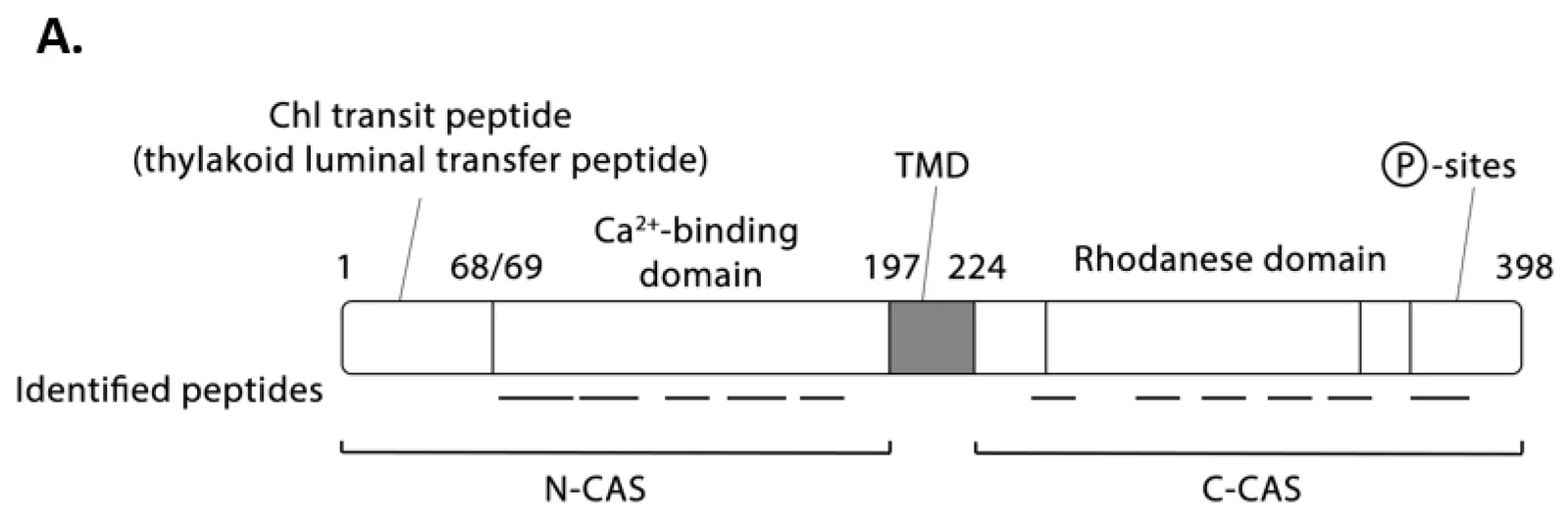

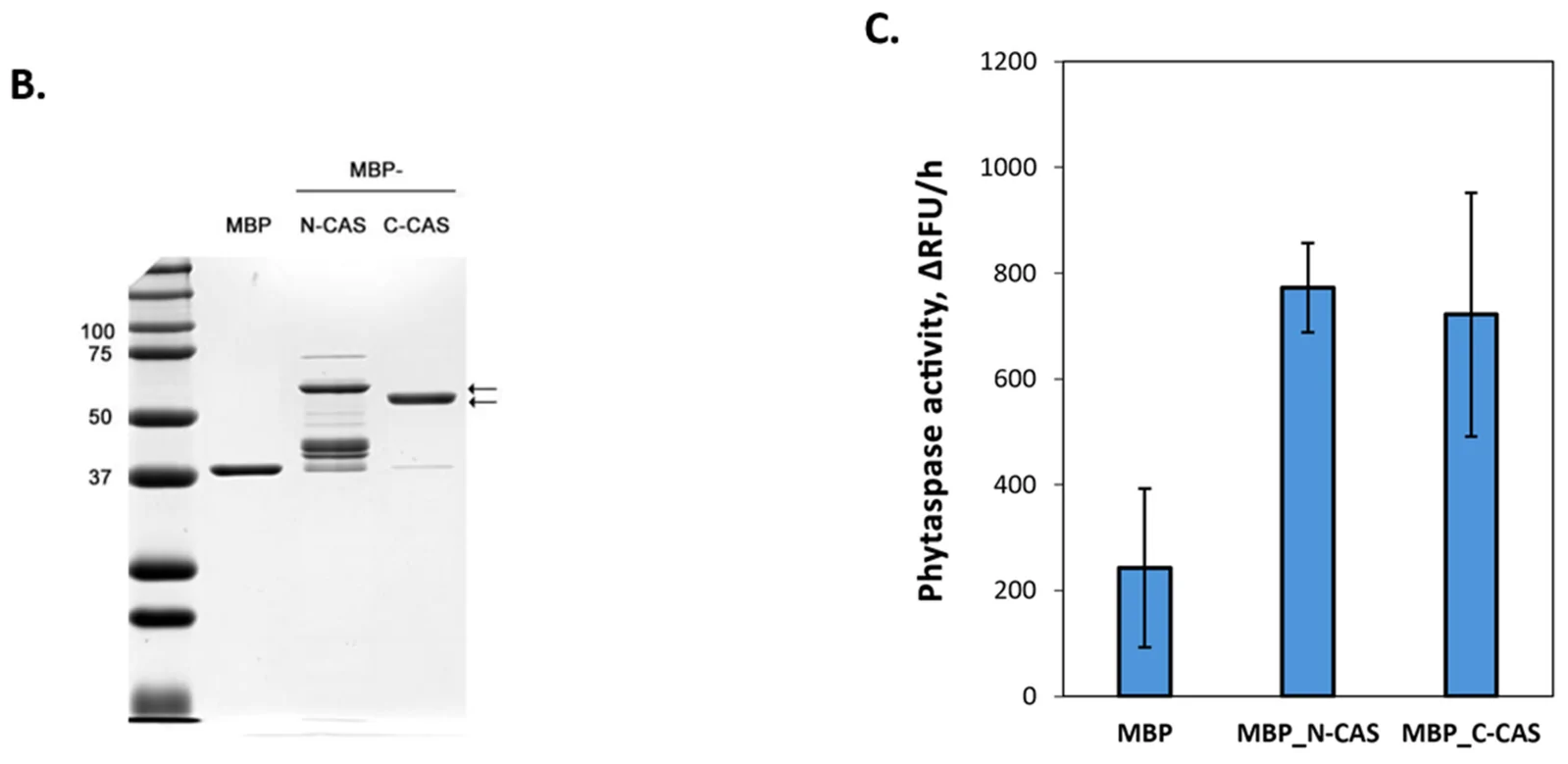
Assessment of the CAS-*Nt*Phyt interaction using purified proteins. (**A**) Schematic representation of plant CAS. The transmembrane domain (TMD) divides CAS into two parts, the N-CAS and C-CAS, of similar length. Ⓟ-sites, phosphorylation sites [19,21,23]. Amino acid numbering is indicated for *Nb*CAS. The positions of tryptic peptides of *Nb*CAS identified by the LC-MS analysis of *Nt*Phyt interactors are underlined. See Supplementary Table S1 for the full list of the identified peptides. (**B**) MBP, MBP_N-CAS, and MBP_C-CAS were overproduced in *E. coli* cells and purified using amylose resin affinity chromatography. Eluates from the resin were analyzed by SDS gel electrophoresis on a 10% gel with Coomassie blue staining. Arrows indicate the positions of intact proteins. (**C**) Equivalent amounts of the amylose resin-immobilized MBP_N-CAS, MBP_C-CAS, and free MBP (approx. 1 µg each) were incubated with *Nt*Phyt (approx. 200 ng). After the elution of the resin-bound proteins with maltose-containing buffer, *Nt*Phyt proteolytic activity was fluorometrically determined in the eluates using 40 µM Ac-VEID-AFC as a phytaspase substrate. Relative rates of hydrolysis were determined as an increase of relative fluorescence units per hour (∆RFU/h). Data represent the mean ± SD of three independent experiments. *p* = 0.0023 for the pair MBP–MBP_N-CAS and *p* = 0.023 for the pair MBP–MBP_C-CAS (one-sided *t*-test with the Benjamini–Hochberg correction).

The initial study localized *At*CAS to the plasma membrane [15], however subsequent analyses pointed to the chloroplast thylakoid membrane as the site of CAS accumulation [17,18,19,20,21]. CAS is an approx. 40 kDa protein with an N-terminal chloroplast transit peptide [18] and a single transmembrane domain in the middle of the protein molecule (Figure 1A). The carboxy terminal half of the protein displays sequence similarity to a noncatalytic rhodanese homology domain, which is probably involved in protein–protein interaction, whereas the amino terminal half of the protein was shown to possess low-affinity/high-capacity Ca^2+^-binding properties (binding 10 to 12 Ca^2+^ per CAS molecule with a K_d_ ~1.2 mM [15]). Thus, plant CAS appears to differ significantly, both in its structure and localization, from animal calcium-sensing receptors (CaSRs), which belong to the cell-surface G-protein-coupled receptor family [22]. Phosphorylation of *At*CAS in the vicinity of its C-terminus in a high light-induced and Ca^2+^-dependent fashion by chloroplast STN7 and STN8 protein kinases was reported [19,21,23].

Available data point to a prominent role of CAS in plant immune signaling in *Arabidopsis*, as immunity was compromised in CAS knockout plants [24]. CAS was shown to be required for both PTI (pathogen-associated molecular pattern (PAMP)-triggered immunity) and ETI (effector-triggered immunity). CAS is involved in PAMP-induced expression of defense genes and acts upstream of salicylic acid accumulation [24]. *Nicotiana benthamiana* plants with silenced CAS display a markedly suppressed hypersensitive response [24] and are more susceptible to virus infection [25,26]. In turn, CAS is targeted by pathogens, and effector proteins HopAU1 of *Pseudomonas syringae* and SsITL of *Sclerotonia sclerotiorum* were reported to interact with CAS [27,28,29]. CAS has also been implicated in abiotic stress responses, such as drought tolerance [30,31].

However, the molecular mechanisms underlying the biological functions of CAS, including its roles in sensing [Ca^2+^]_ext_, participation in stress responses, plant immunity, and cell death remain largely unknown. As the interaction of CAS with phytaspase might provide new clues to the functioning of both proteins, we strove to elucidate when and where this interaction could take place. Data presented in this study favor a model in which the CAS–phytaspase interaction likely occurs before CAS reaches its final destination in the chloroplasts, possibly at the plasma membrane, with implications for CAS trafficking. On the other hand, we also uncovered a requirement for CAS for stress-induced retrograde transport of the cell death-related protease, *Nt*Phyt.

## 2. Results

### 2.1. Identification of CAS as a Phytaspase Interactor

In order to elucidate the molecular mechanisms of *Nt*Phyt functioning, a search for *Nt*Phyt-interacting proteins was performed using the proximity-dependent biotin-based identification approach. The TurboID biotin ligase was fused to the C-terminus of *Nt*Phyt (*Nt*Phyt-TurboID), and the recombinant protein was produced in *N. benthamiana* leaves via agroinfiltration. Production of the TurboID attached to the signal peptide (SP) of *Nt*Phyt (SP-TurboID) served as a control. Leaves producing each of the proteins were infiltrated with biotin; biotinylated proteins were then isolated from both types of leaves by affinity chromatography using Streptavidin Magnetic Beads, and tryptic peptides thereof were analyzed using a liquid chromatography–mass spectrometry (LC-MS) approach.

The LC-MS identification yielded far more candidate *Nt*Phyt interactors than the approach that we used previously, which utilized electrophoretic separation of the biotinylated proteins followed by MS-based identification of bands of interest [13]. Among numerous proteins enriched in the *Nt*Phyt-TurboID samples (versus the SP-TurboID ones), the *N. benthamiana* Calcium-sensing receptor (*Nb*CAS) was identified (NbD051503.1; GenBank: BAM66423.1). Over 10 tryptic peptides of *Nb*CAS were characterized (Figure 1A and Supplementary Table S1) allowing the reliable identification of the protein.

To verify the results of the in vivo BioID analysis, the possibility of a direct in vitro interaction between purified *Nt*Phyt and CAS was assessed. As CAS consists of two domains, the N-terminal (N-CAS) and the C-terminal (C-CAS), separated by the transmembrane domain (TMD; Figure 1A), two halves of *Nt*CAS were produced in *E. coli* cells as MBP (maltose-binding protein) fusion proteins, MBP_N-CAS and MBP_C-CAS, and immobilized on the Amylose resin (Figure 1B). Free MBP protein served as a negative control. Binding of *Nt*Phyt to these probes was assessed by using *Nt*Phyt-His_6_ isolated from the apoplast of *N. benthamiana* leaves via Ni-NTA agarose affinity chromatography. Upon incubation of the immobilized CAS derivatives with *Nt*Phyt, the unbound material was discarded, the MBP-containing proteins were eluted from the resin with maltose, and the proteolytic activity of *Nt*Phyt was determined fluorimetrically in the eluates using the *Nt*Phyt-specific fluorogenic peptide substrate, Ac-VEID-AFC [7] (Figure 1C). Somewhat unexpectedly, both halves of the CAS protein bound to *Nt*Phyt.

The results indicate that *Nt*Phyt is able to interact with CAS, as revealed by the in vivo and in vitro assays.

### 2.2. CAS Is a Phytaspase Substrate

**Figure 2:**
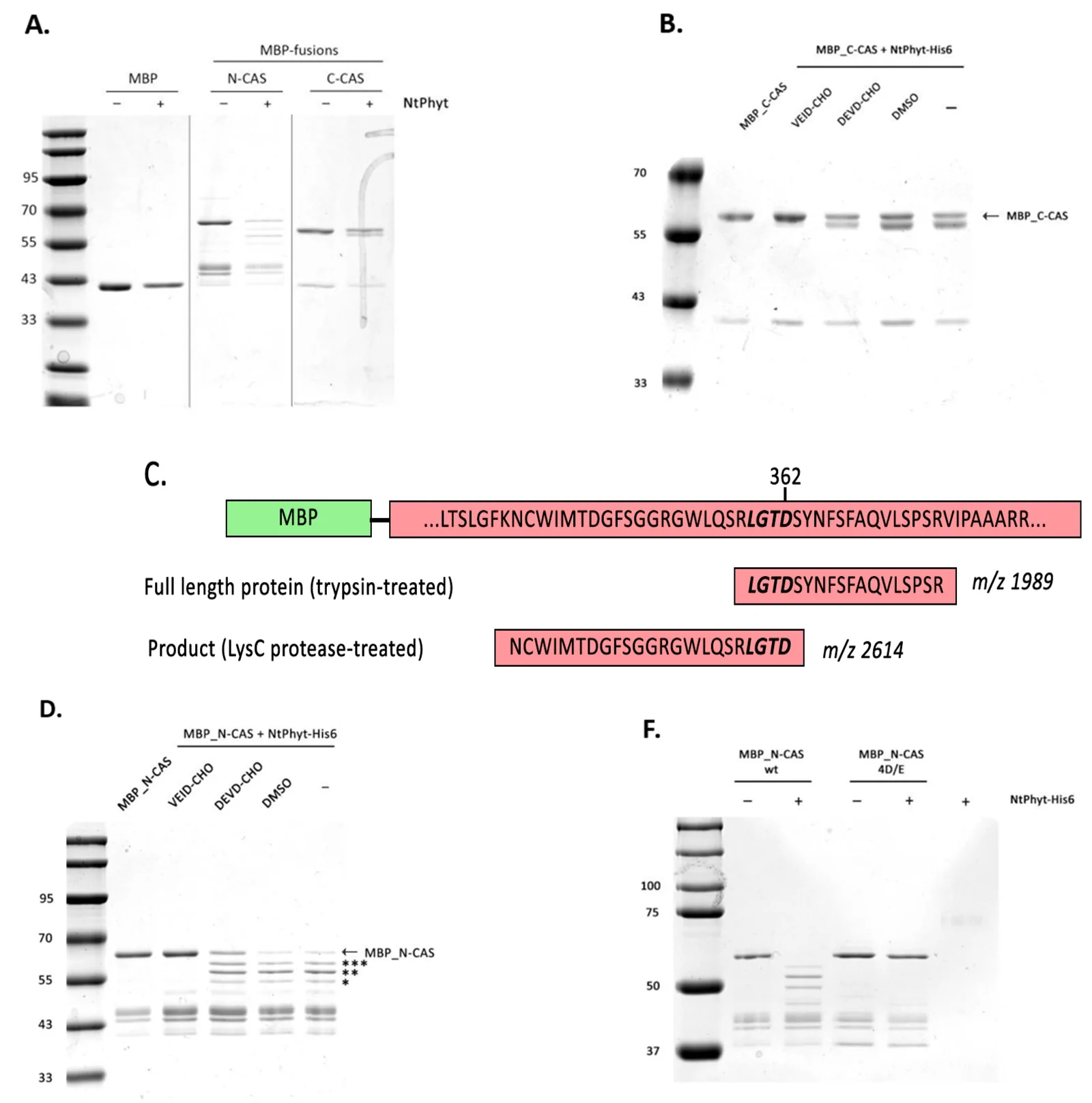

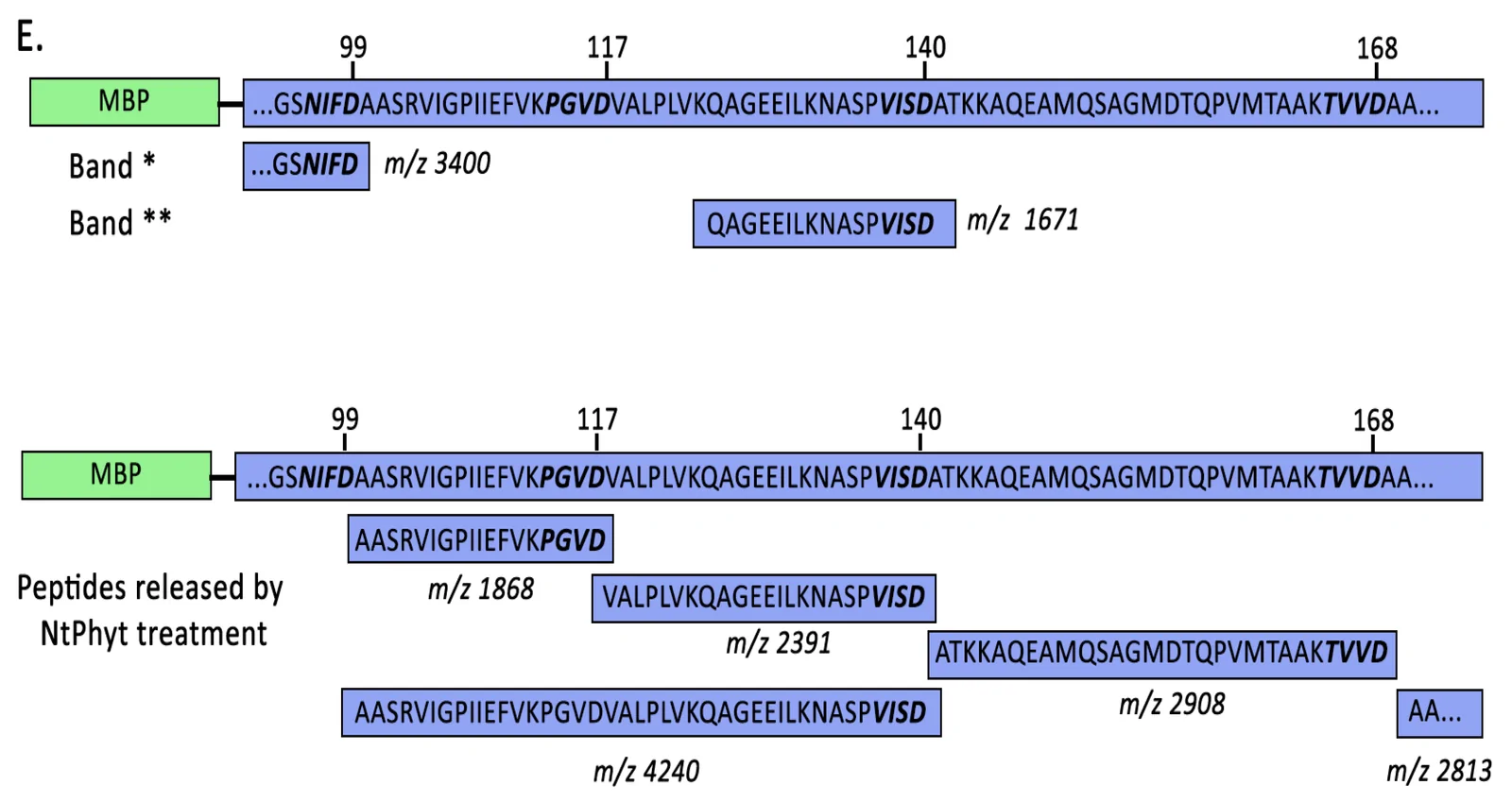
CAS is a substrate of *Nt*Phyt. (**A**) Treatment of the immobilized MBP, MBP_N-CAS, and MBP_C-CAS with *Nt*Phyt (Phyt+), relative to untreated samples (Phyt−), results in fragmentation of CAS derivatives (but not of free MBP). (**B**,**D**) Hydrolysis was suppressed by Ac-VEID-CHO, but not by Ac-DEVD-CHO. Both inhibitors were used at a concentration of 100 µM. In (**A**,**B**,**D**,**F**), reaction products were fractionated by 10% SDS gel electrophoresis and stained with Coomassie blue. (**C**) For MBP_C-CAS, MS analysis of the product (lower band in (**B**)) revealed a characteristic semi-LysC peptide (*m*/*z* 2614) pointing to cleavage at D362, whereas in the full-length protein (upper band in (**B**)) this region is intact (a characteristic tryptic peptide with *m*/*z* 1985). (**E**) Schematic representation of *Nt*Phyt cleavage products of MBP_N-CAS. MS analyses revealed characteristic semi-tryptic peptides with *m*/*z* 3400 and 1671 derived from the (*) and (**) product bands in (**D**), respectively. For band (***), identification of the cleavage site required analysis of the peptides released from the resin by *Nt*Phyt treatment due to the proximity of the Lys residue to the cleavage site at D168. Note that the released peptides were not treated with trypsin and therefore represent peptides generated by a pair of *Nt*Phyt cleavages: at D99 and D117 (*m*/*z* 1868), at D117 and D140 (*m*/*z* 2391), at D99 and D140 (*m*/*z* 4240), and at D140 and D168 (*m*/*z* 2908). (**F**) The MBP_N-CAS 4D/E mutant is phytaspase-resistant.

Comparison of the MBP_N-CAS, MBP_C-CAS, and free MBP samples eluted from the Amylose resin either before or after incubation with *Nt*Phyt revealed that treatment with *Nt*Phyt resulted in fragmentation of both MBP_N-CAS and MBP_C-CAS, whereas free MBP remained intact (Figure 2A). Preincubation of *Nt*Phyt with its specific inhibitor Ac-VEID-CHO [7] prior to the addition of the enzyme to the immobilized MBP_N-CAS and MBP_C-CAS proteins prevented CAS fragmentation (Figure 2B,D), whereas preincubation of *Nt*Phyt with the caspase-3-specific inhibitor Ac-DEVD-CHO had no effect. The results demonstrate that CAS fragmentation occurs in a *Nt*Phyt-specific manner. Interestingly, the presence of several cleavage sites in N-CAS is rather unusual for a highly specific protease such as *Nt*Phyt.

To identify the single *Nt*Phyt cleavage site in the MBP_C-CAS protein, MALDI-TOF mass spectrometry analyses of the tryptic and endoproteinase LysC hydrolysates were performed using the full-length protein band and the truncated product band (Figure 2B), respectively. These analyses revealed that fragmentation of MBP_C-CAS occurred, consistent with the substrate specificity of *Nt*Phyt, after the Asp residue in the LGTD^362^ motif (Figure 2C).

For the MBP_N-CAS *Nt*Phyt cleavage products, MALDI-TOF analysis of tryptic hydrolysates of proteins from the individual product bands (marked as *, **, and *** in Figure 2D) was only partially successful. This analysis revealed that band (*) was generated by cleavage after the Asp residue in the NIFD^99^ motif, whereas band (**) resulted from cleavage at the VISD^140^ motif (Figure 2E). No characteristic semi-tryptic peptide could be detected for band (***), probably because of the proximity of a Lys residue to the *Nt*Phyt cleavage site. To overcome this difficulty, we performed MALDI-TOF analysis of the peptides released into solution from immobilized MBP_N-CAS by *Nt*Phyt treatment (Figure 2E). This analysis confirmed the presence of characteristic peptides generated by cleavage at the NIFD^99^ and VISD^140^ motifs (see the figure legend for a detailed description). It also allowed us to identify the cleavage site at KTVVD^168^, which generated product band (***) and had escaped identification in the previous analysis. Interestingly, an additional *Nt*Phyt cleavage site, PGVD^117^, was also identified. However, hydrolysis at this site was the least efficient, as indicated by the presence of an overlapping peptide with an *m*/*z* of 4240 and the absence of a corresponding band in Figure 2D.

To ensure that no *Nt*Phyt cleavage sites in the MBP_N-CAS protein had been missed, the Asp (D) residues at the identified cleavage sites were mutated to Glu (E), generating MBP_N-CAS-4D/E (*Nt*Phyt does not cleave after Glu [8]). The resulting mutant protein was resistant to *Nt*Phyt treatment (Figure 2F).

We concluded from these studies that *Nt*Phyt is able not only to associate with the N- and C-domains of CAS, but also to introduce specific cleavages into both halves of the CAS molecule, at least in vitro. Notably, comparison of amino acid sequences of CAS proteins from distantly related plant species for the presence of the identified phytaspase cleavage sites revealed that most of these sites are relatively well conserved during evolution (Supplementary Table S2).

### 2.3. NtCAS, but Not Phytaspase, Is Present in Intact Chloroplasts

**Figure 3:**
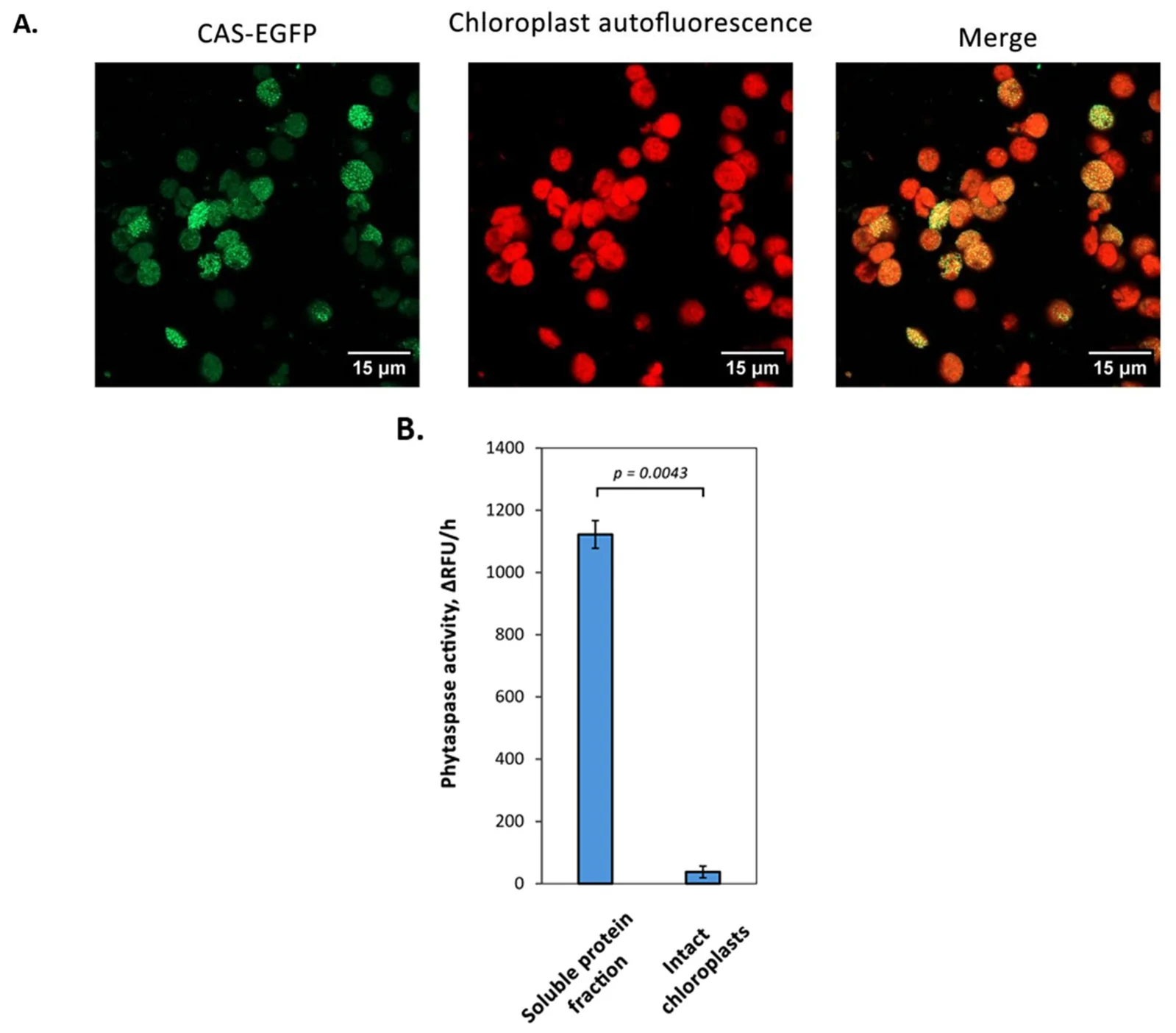
*Nt*CAS, but not phytaspase, is present in intact *N. benthamiana* chloroplasts. (**A**) Confocal microscopy of intact chloroplasts isolated from *N. benthamiana* leaves producing *Nt*CAS-EGFP. (**Left image**), *Nt*CAS-EGFP fluorescence; (**central image**), chloroplast autofluorescence; (**right image**), merge. (**B**) Phytaspase proteolytic activity was fluorimetrically determined in equal aliquots of the soluble protein fraction of crude leaf extract and of the lysate of isolated chloroplasts using 40 µM Ac-VEID-AFC as a phytaspase substrate. The mean ± SD of two independent experiments, with three technical replications in each, is presented. *p*-value is indicated (the Wilcoxon rank-sum test, two-sided).

Available data indicate that *At*CAS accumulates in the thylakoid membrane of chloroplasts [17,18,19,20,21]. To assess whether the *Nt*CAS localizes similarly, the *Nt*CAS-EGFP protein was produced in *N. benthamiana* plants via agroinfiltration, and a fraction of intact chloroplasts was purified from leaf extract via differential centrifugation. Fluorescence microscopy examination of the sample indeed revealed the presence of *Nt*CAS-EGFP fluorescence in intact chloroplasts (Figure 3A).

We then compared the distribution of phytaspase-specific proteolytic activity between the chloroplasts and the water-soluble fractions of *N. benthamiana* leaves using the Ac-VEID-AFC fluorogenic *Nt*Phyt substrate. Whereas *Nt*Phyt activity was abundant in the soluble protein fraction, it was undetectable in the lysate of the intact chloroplasts (Figure 3B).

Our analysis thus indicates that *Nt*CAS, like its *At*CAS counterpart, accumulates in the chloroplasts of *N. benthamiana* leaves. However, phytaspase activity is absent in the chloroplasts, and therefore an interaction of CAS with *Nt*Phyt might be expected to occur before *Nt*CAS reaches its final chloroplastic destination.

### 2.4. Dual Localization of NtCAS in N. benthamiana Leaf Cells

**Figure 4:**
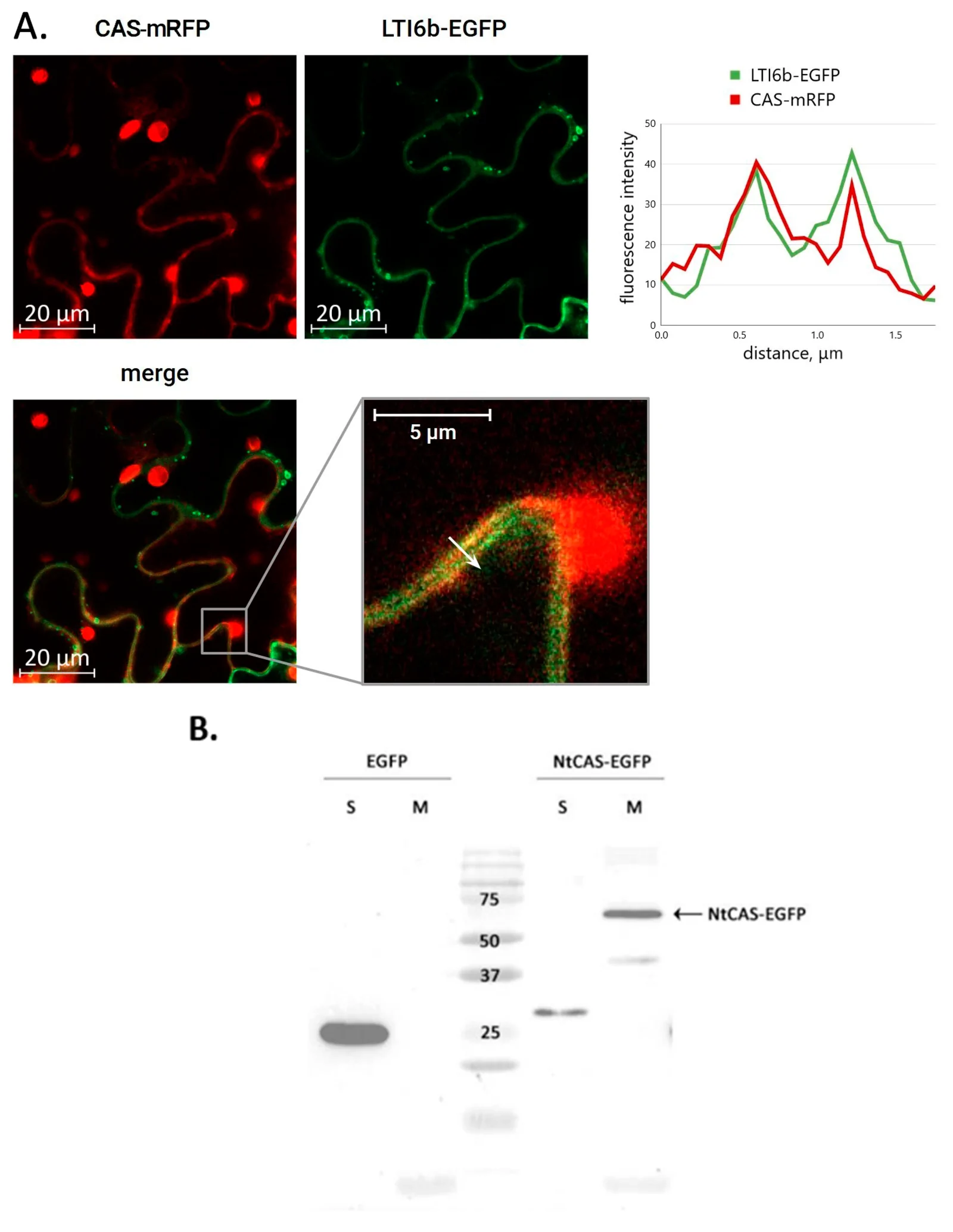

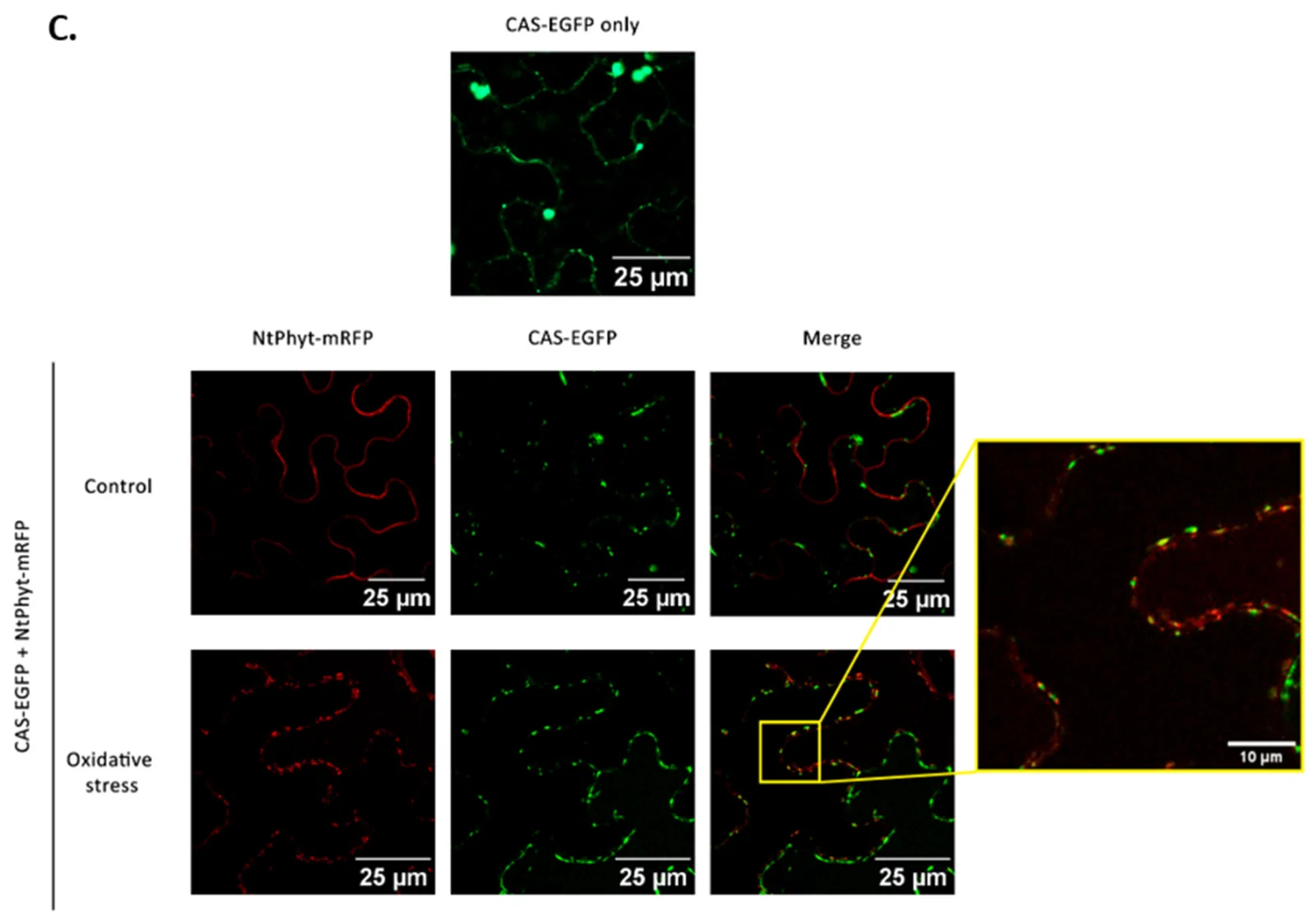
CAS localization in *N. benthamiana* cells. (**A**) Confocal microscopy of *Nt*CAS-mRFP and LTI6b-EGFP co-produced in *N. benthamiana* leaf cells by agroinfiltration at 2 dpi. Diagram on the right: measurement of green (LTI6b-EGFP) and red (*Nt*CAS-mRFP) fluorescence intensities across the boundaries of the adjacent cells (shown by the arrow in the merged image). (**B**) The water-soluble (s) and membrane protein (m) fractions from leaves producing either *Nt*CAS-EGFP or free EGFP were separated by 10% SDS-PAGE. Western blot (WB) analysis was performed using an anti-EGFP antibody. (**C**) Confocal fluorescence microscopy visualization of *Nt*Phyt-mRFP and *Nt*CAS-EGFP co-produced in *N. benthamiana* leaves reveals differential localization of the proteins in the non-stressed tissue (upper row) and partial co-localization of the two proteins upon the induction of oxidative stress (treatment with 50 µM antimycin A for 11 h, lower row). Microscopy visualization of cells producing CAS-EGFP only is provided above for comparison.

To learn whether the chloroplasts are the only site of *Nt*CAS localization, *N. benthamiana* leaves producing the *Nt*CAS-mRFP protein were inspected using fluorescence microscopy. Alongside a clear signal in the chloroplasts, peripheral localization of *Nt*CAS-mRFP was also observed (Figure 4A), which co-localized with the plasma membrane marker protein LTI6b-EGFP [32] (Figure 4A), see the diagram on the right.

Separation of extracts from *Nt*CAS-EGFP-producing leaves into water-soluble and insoluble protein fractions revealed the presence of the intact *Nt*CAS-EGFP in the fraction of insoluble proteins (Figure 4B), consistent with the presence of a transmembrane domain in the middle of the protein molecule (Figure 1A).

To compare localization of *Nt*Phyt and *Nt*CAS directly, *Nt*Phyt-mRFP and *Nt*CAS-EGFP were co-produced in *N. benthamiana* leaves. Under normal conditions, no clear co-localization of the proteins was observed (Figure 4C, the upper row), although overproduction of *Nt*Phyt apparently weakened the CAS signal and imparted a more ‘dotted’ localization to CAS-EGFP (Figure 4C). Upon the induction of oxidative stress by treatment with antimycin A, both the retrograde transport of *Nt*Phyt and partial co-localization of *Nt*CAS with *Nt*Phyt within the structures located in the vicinity of cell borders were observed (Figure 4C, the lower row). These results suggest that triggering *Nt*Phyt re-import into the cell may increase its chances of encountering CAS.

### 2.5. CAS Precursor Protein—A New Player

**Figure 5:**
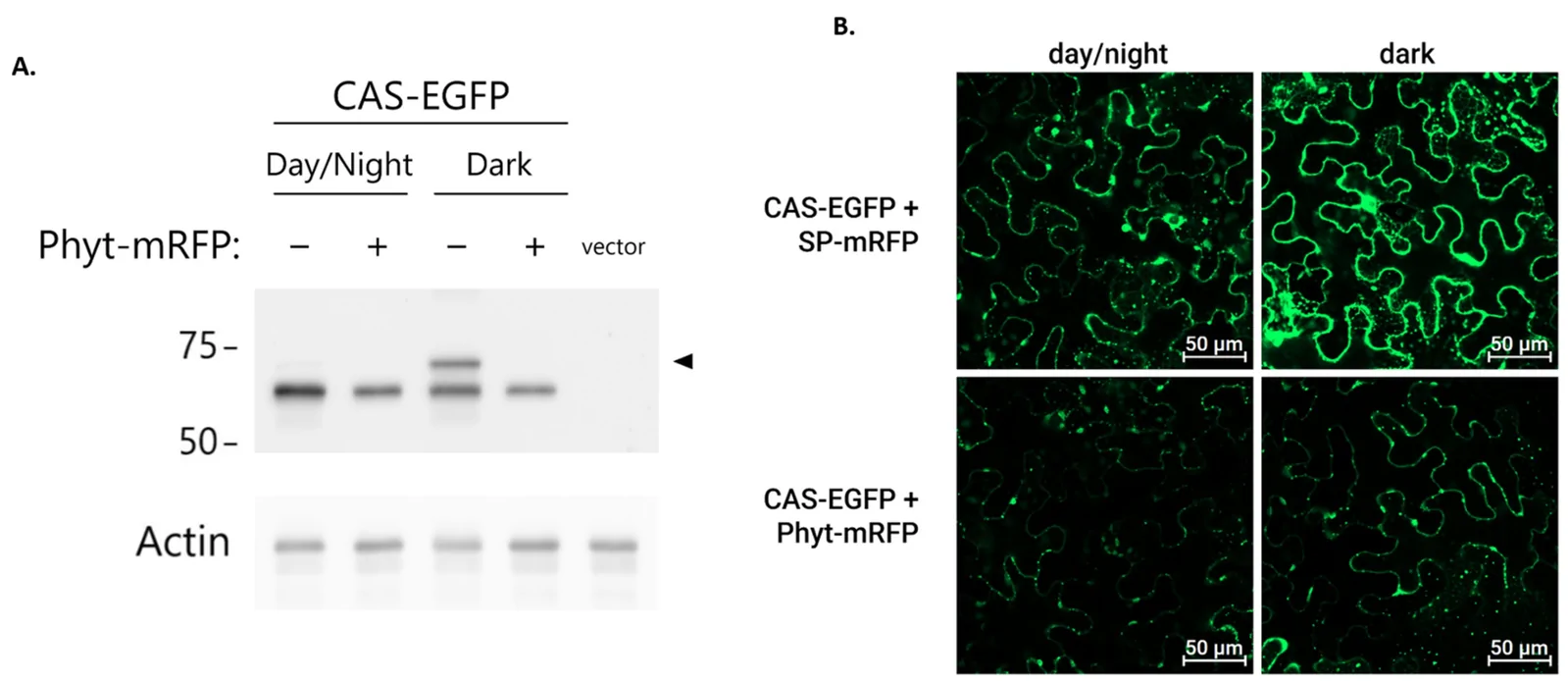
Dark-induced accumulation of the ~70 kDa *Nt*CAS-EGFP form correlates with the peripheral localization of the CAS protein in *N. benthamiana* cells and is sensitive to increased *Nt*Phyt levels. *Nt*CAS-EGFP was co-produced in *N. benthamiana* leaves with either SP-mRFP or *Nt*Phyt-mRFP under the normal illumination (Day/Night) or upon incubation of plants in the dark for 2 days (dark). SP-mRFP, an apoplast-targeted version of mRFP [12] was used as a control for *Nt*Phyt-mRFP production. (**A**) Proteins were separated by 10% SDS-PAGE. WB analysis was performed using an anti-EGFP antibody. The preCAS-EGFP band is indicated with the triangle. Lower panel: loading control with anti-actin antibodies. (**B**) Confocal fluorescence microscopy examination of the leaf samples. The images were obtained using identical microscope settings.

Western blot analysis of *Nt*CAS-EGFP produced in *N. benthamiana* leaves revealed CAS as a major protein band of approximately 60 kDa. Apart from the presence of some degradation products with lower masses, a faint protein band of approximately 70 kDa was also visible (Figure 5A). This slower-migrating band most likely corresponds to the CAS-EGFP precursor protein (calculated MW ~70.3 kDa) with the N-terminal chloroplast-targeting sequence not yet cleaved. According to SignalP 6.0 prediction, the N-terminal region (68 amino acid residues long) of the full-length *Nt*CAS encompasses a chloroplast transit peptide/thylakoid luminal transfer peptide, expected to be cleaved off upon the import of CAS into the chloroplasts. Therefore, the 70 kDa protein (named preCAS-EGFP hereafter) likely represents the form of CAS that has not yet been delivered to the chloroplasts.

We noticed that the preCAS-EGFP band was significantly enhanced, at the expense of the mature 60 kDa protein band, upon prolonged (2 days) incubation of plants in the dark (Figure 5A). Consistently, fluorescence microscopy analysis revealed an enhanced peripheral localization of *Nt*CAS-EGFP in the dark-incubated leaves (Figure 5B).

Co-production of *Nt*Phyt-mRFP with *Nt*CAS-EGFP moderately decreased the intensity of the processed *Nt*CAS-EGFP band, irrespective of illumination conditions (Figure 5A). Notably, in the samples from dark-incubated leaves, the preCAS-EGFP band was completely eliminated upon *Nt*Phyt production (Figure 5A).

We interpret these findings to indicate that the *Nt*Phyt level is able to influence the behavior of CAS in vivo and that the preCAS, rather than the chloroplast-localized mature CAS, may be competent for interaction with *Nt*Phyt.

### 2.6. CAS Is Required for Stress-Induced Internalization of Phytaspase

**Figure 6:**
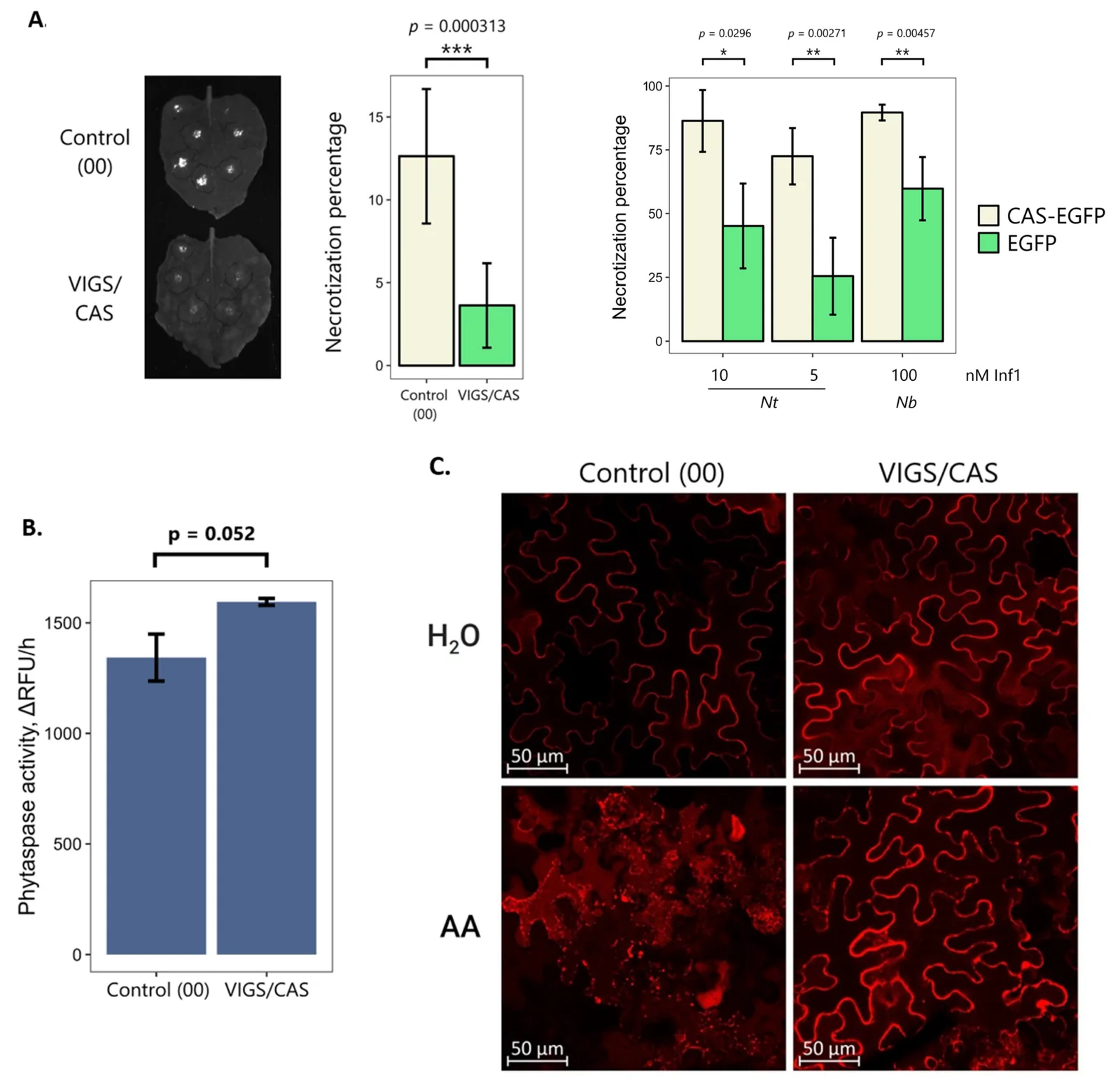
Down-regulation of *Nb*CAS suppresses stress-induced internalization of *Nt*Phyt. (**A**) INF1-induced cell death is suppressed in the *Nb*CAS-silenced plants (VIGS/CAS), relative to the empty virus control (00). INF1 (100 nM) was infiltrated into leaves, and photographs were taken under UV light (365 nm) 2 days later. The areas of necrosis were measured using ImageJ version 1.54 (Fiji). Overproduction of *Nt*CAS-EGFP (**right panel**), in contrast, enhanced the INF1-induced necrotization in *N. benthamiana* (*Nb*) and *N. tabacum* (*Nt*) leaves (relative to production of free EGFP). (**B**) Proteolytic activity of endogenous phytaspase does not differ significantly in the CAS-silenced and control *N. benthamiana* plants. Phytaspase activity was determined in leaf extracts using 40 µM Ac-VEID-AFC as a substrate. (**C**) (**Upper row)**: *Nt*Phyt-mRFP localization in the CAS-silenced and control plants under normal conditions. (**Lower row)**: Induction of oxidative stress via leaf treatment with 50 µM antimycin A (AA) causes *Nt*Phyt-mRFP internalization in control plants. In the CAS-silenced plants, however, *Nt*Phyt-mRFP retains apoplastic localization despite the antimycin A treatment. Statistical evaluation: 5–6 independent biological replicates for VIGS experiments, 3–5 independent biological replicates for CAS-EGFP/Inf experiments. For phytaspase activity measurement, data are presented as means ± SD from three independent biological replicates (different plants, *n* = 3). Each experiment was independently repeated at least three times with similar results. Statistical significance was analyzed using a *t*-test,*—*p* < 0.05, **—*p* < 0.01, ***—*p* < 0.001.

To learn whether, in turn, the CAS protein may be involved in *Nt*Phyt functioning, we compared *Nt*Phyt-mRFP behavior in the *Nb*CAS-silenced plants with that in the control plants. Tobacco rattle virus (TRV) derivatives targeting two distinct regions of *N. benthamiana* CAS mRNA were constructed. Approximately a five- to ten-fold reduction in the *Nb*CAS mRNA level was achieved with both constructs in virus-infected leaves (Supplementary Figure S1), and similar functional results were obtained. Functionally, the CAS-silenced plants displayed a significantly suppressed necrotization upon challenge with INF1 protein of *Phytophthora infestans* (Figure 6A), as expected [24]. Overproduction of CAS-EGFP, in contrast, enhanced the INF1-induced necrotization in *N. benthamiana* (*Nb*) and *N. tabacum* (*Nt*) leaves (Figure 6A, right panel). Interestingly, judging by the DAB (3,3′-diaminobenzidine) staining, the antimycin A-induced generation of reactive oxygen species (ROS) [33,34] apparently was not impaired in the CAS-silenced plants (Supplementary Figure S2).

Under normal conditions, suppression of the CAS production did not significantly affect the level of *Nt*Phyt-specific proteolytic activity in the leaves (Figure 6B) and appeared to exert no effect on the secretion of *Nt*Phyt-mRFP into the apoplast (Figure 6C). Upon the induction of oxidative stress via antimycin A treatment, retrograde transport of *Nt*Phyt became detectable in the control leaves, as indicated by the formation of dotted structures in the vicinity of the plasma membrane and inside the cell. In the CAS-silenced plants, however, treatment with antimycin A failed to induce *Nt*Phyt re-import into the cell (Figure 6C). A smooth apoplastic localization of *Nt*Phyt was preserved, indicating that its internalization was suppressed.

Thus, this result uncovers a requirement for CAS in stress-induced retrograde transport of *Nt*Phyt.

## 3. Discussion

The results presented in this study point to an unexpected link between tobacco phytaspase, a plant cell death-related protease with predominantly apoplastic localization, and the calcium-sensing receptor (CAS), a mediator of the external Ca^2+^-induced cytosolic Ca^2+^ increase in plants. *At*CAS was initially identified as a plasma membrane protein [15], although numerous subsequent studies point to the thylakoid membrane of chloroplasts as the site of CAS accumulation [17,18,19,20,21].

### 3.1. Evidence Supporting a Possible Interaction Between CAS and Phytaspase

Here, we present evidence that *Nt*Phyt is absent from the *N. benthamiana* chloroplasts. Yet, despite the clear difference in the localization of *Nt*CAS and *Nt*Phyt, the following arguments appear to support the possibility of an interaction between the two proteins. (1) CAS was identified as a candidate *Nt*Phyt interactor in the in vivo BioID search; (2) direct interaction (binding) between *Nt*CAS and *Nt*Phyt was demonstrated in vitro using the purified proteins; (3) *Nt*Phyt was able to specifically cleave *Nt*CAS at several sites in vitro; (4) dual localization of *Nt*CAS, both in chloroplasts and at the cell periphery, including the plasma membrane, was observed in *N. benthamiana* cells overproducing *Nt*CAS-mRFP; (5) partial co-localization of *Nt*CAS with *Nt*Phyt was observed in cells subjected to oxidative stress; (6) *Nt*CAS is required for stress-induced retrograde transport of *Nt*Phyt; and (7) a CAS precursor protein that apparently retains the chloroplast-targeting signal (preCAS) was shown to accumulate upon prolonged incubation of plants in the dark and to disappear in the presence of elevated *Nt*Phyt levels.

Of course, each of these arguments can be individually critically addressed. But collectively, these observations appear to provide grounds for considering the potential consequences of the CAS-Phyt interaction. An obvious first question is where *Nt*CAS and *Nt*Phyt could meet. *Nt*Phyt provides an example of how protein trafficking in plant cells may be a rather complex and multistep process. Indeed, phytaspase is synthesized as an inactive precursor protein, traverses the endoplasmic reticulum and the Golgi apparatus, and becomes autocatalytically processed in the course of anterograde transport to the apoplast. However, upon perception of cell death-inducing stimuli, *Nt*Phyt utilizes the clathrin-mediated endocytosis to re-enter plant cells [7,11,12].

To the best of our knowledge, the route taken by CAS to reach the thylakoid membrane of chloroplasts has not been addressed in detail. Therefore, in principle, the interaction of CAS with *Nt*Phyt could be proposed to occur at any stage of the phytaspase trafficking route. Of these many possibilities, we would like to consider a scenario, consistent with our confocal fluorescence microscopy observations, proposing that CAS transiently localizes to the plasma membrane as an intermediate location on its way to its final destination, the chloroplasts.

### 3.2. Could There Be a Rationale for Transient Plasma Membrane Localization of CAS?

It is presumably at this location that CAS is trapped upon prolonged incubation of plants in the dark, judging by the accumulation of the preCAS form and enhanced peripheral localization in dark-incubated plants (Figure 5). As CAS possesses a single transmembrane domain in the middle of the molecule, its plasma membrane localization would result in the exposure of one of its soluble domains to the apoplast, thus allowing interaction with the apoplastic *Nt*Phyt. Notably, *Nt*Phyt-dependent elimination of preCAS, but not of the mature CAS protein, was observed in dark-incubated plants (Figure 5).

Transient localization of CAS at the plasma membrane could be consistent with the requirement for CAS in *Nt*Phyt internalization, as shown in this study. On the other hand, a mechanism allowing thylakoid membrane-localized CAS to sense [Ca^2+^]_ext_ is intriguing but unknown. Transient localization of preCAS at the plasma membrane could contribute to solving this problem.

Also of note, a trafficking pathway linking the plasma membrane and chloroplasts has been described for a number of proteins of plant and plant pathogen origin, which re-localize from the plasma membrane to chloroplasts upon activation of plant defense responses [25]. This mobility relies on the co-existence of two subcellular targeting signals in their molecules, an N-terminal myristoylation site and a chloroplast transit peptide. As CAS possesses a chloroplast transit peptide but lacks a myristoylation site, its delivery to the plasma membrane should occur in a different way, possibly through binding to a plasma membrane-localized CAS interactor.

### 3.3. Phytaspase-Mediated Fragmentation of CAS

The mode of *Nt*CAS fragmentation by *Nt*Phyt also deserves attention. Based on our previous studies of phytaspases, these are proteases of exceptional selectivity. Only a few protein substrates of phytaspases have been discovered to date, and typically, phytaspases introduce only one or two cleavages in their targets, in vitro as well as in vivo [7,35,36,37]. The identification of five *Nt*Phyt cleavage sites in CAS therefore came as a surprise. The need to introduce as many as four cleavages into the N-terminal Ca^2+^-binding domain of CAS is enigmatic. Of note, these cleavage site motifs are relatively well conserved in the CAS proteins of various plant species.

The data presented in this study indicate that *Nt*CAS and *Nt*Phyt are likely to interact even under non-stress conditions, although the interaction might be enhanced upon stress induction that triggers retrograde transport of *Nt*Phyt. Partial co-localization of *Nt*CAS with *Nt*Phyt in stressed cells implies that the *Nt*Phyt-mediated CAS fragmentation may start while preCAS is localized at the plasma membrane and proceed upon *Nt*Phyt internalization.

A role for [Ca^2+^] regulation in a plethora of processes related to life and death decisions of plant cells is broadly acknowledged [38,39,40]. In relation to plant cell death, for instance, an increase in intracellular [Ca^2+^] in the course of the hypersensitive response to pathogens is known to trigger a ROS burst that promotes the death of infected cells [41,42,43,44]. Could the *Nt*Phyt-mediated fragmentation of CAS function to disturb the [Ca^2+^]_cyt_ balance and be a part of the plant cell death program, thereby ensuring the immune response in plants? We think that this and several other important questions arising from this study deserve further elucidation.

## 4. Materials and Methods

### 4.1. Plant Growth Conditions and Agroinfiltration

*Nicotiana benthamiana* plants were grown at 25 °C in soil in a controlled environment under a 16 h/8 h day/night cycle and 65% relative humidity. For transient protein production, *Agrobacterium tumefaciens* C58C1 cells were transformed with the respective plasmid. Agrobacterium suspensions (OD_600_ = 0.8–1.0) in MMA buffer (10 mM MES, pH 5.7, 10 mM MgCl_2_ and 100 μM acetosyringone) were infiltrated into leaves of 6-week-old plants using a blunt syringe. Typically, leaves were harvested 2 days post-infiltration (dpi), unless otherwise indicated in the figure legends.

### 4.2. Plasmid Construction

Construction of the *Nt*Phyt-TurboID and SP-TurboID genes within the pLEX7000 expression vector [36] was described previously [13]. The ORF of *N. tabacum* CAS protein (approx. 1200 bp) was amplified by PCR using total *N. tabacum* DNA and primers CAS_Kpn_Nco_dir and CAS_Bam_rev (see Supplementary Table S3 for primer sequences). The PCR product was cloned into the pLEX7000 expression vector upstream of and in frame with the EGFP gene to generate the pLEX_NtCAS-EGFP plasmid. The DNA fragments encoding N-CAS and C-CAS were PCR-amplified using the CAS_Head_Bam_dir plus CAS_Head_Sac_rev (for the N-terminal half of CAS) and CAS_Tail_Bam_dir plus CAS_Tail_Sac-rev (for the C-terminal half) primer pairs, respectively, and inserted downstream of and in frame with the MBP-coding sequence in the pET28a_MBP plasmid [14]. To obtain the MBP_N-CAS-4(D/E) mutant, mutations were sequentially introduced via megaprimer PCR using mutagenizing primers CAS2_D99E_dir, CAS2_D117E_rev, CAS2_D140E_dir, and CAS2_D168E_dir.

To construct TRV2 derivatives for VIGS, cDNA fragments of *N. benthamiana* CAS were PCR-amplified using NbCAS3_VIGS_Kpn_dir plus NbCAS3_VIGS_Bam_rev and NbCAS3_ORFVIGS_Kpn_dir plus NbCAS3_ORFVIGS_Bam_rev primer pairs, and DNA products were ligated between the corresponding sites of the pYL156 TRV2 vector [45,46] to produce the TRV2:NbCAS_UTR and TRV2:NbCAS_ORF plasmids, respectively.

The identities of all constructs were confirmed by DNA sequence analysis. Bacterial and plant expression of the constructed protein-encoding genes was driven by the T7 promoter and the dual cauliflower mosaic virus (*CaMV*) 35S promoter, respectively.

### 4.3. Transient Expression in Plants and Protein Fractionation

Leaves of *N. benthamiana* plants were infiltrated with agrobacteria carrying the constructed binary plasmids in combinations described in the figure legends. At 2 dpi, leaf discs (approx. 15 mg each) from plants transiently producing the recombinant proteins were prepared and examined using confocal fluorescence microscopy. Where indicated, leaf discs were vacuum infiltrated with water containing 50 μM antimycin A (Sigma, St. Louis, MO, USA; from a stock solution in ethanol) at a reduced pressure of 30 hPa (mbar) for 1 min and incubated for 4 h to overnight (depending on the concentration of antimycin A used) in the dark prior to microscopic examination. Control discs were infiltrated with distilled water supplemented with an equivalent amount of ethanol.

Isolation of chloroplasts from *N. benthamiana* leaves (approximately 20 g) transiently producing the CAS-EGFP protein for 2 days was performed according to [47]. Briefly, leaves were homogenized in Grinding medium (0.33 M sucrose, 30 mM pyrophosphate, pH 7.8, BSA (0.1% *w*/*v* final)) using a mechanized razor-blade chopper. The resulting homogenate was filtered through Miracloth and centrifuged for 10 min at 1200× *g* at 4 °C. The pellet was resuspended in washing medium (0.33 M sucrose, 20 mM MOPS, pH 7.8) and loaded on the discontinuous 40/80% (*v*/*v*) Percoll gradient. After centrifugation for 20 min at 3000× *g* at 4 °C, the fraction of intact chloroplasts was recovered at the bottom interface, washed with washing medium, and examined by fluorescence confocal microscopy. For phytaspase activity determination, the isolated chloroplasts were resuspended in B1 buffer (50 mM MES, pH 5.7, 50 mM NaCl, 2 mM DTT, 0.1% Tween 20, and 5% glycerol) supplemented with 1.5% DDM (n-dodecyl-β-D-maltopyranoside, dodecyl maltoside; Anatrace, Maumee, OH, USA) [48] and subjected to three freeze–thaw cycles using liquid nitrogen. The chloroplast lysates were diluted two-fold with B1 buffer prior to phytaspase activity measurement.

For protein fractionation, leaf material was frozen in liquid nitrogen and disrupted in a Minilys homogenizer (Bertin Instruments, Montigny-le-Bretonneux, France) using 1.4 mm zirconium oxide beads by five 5 s bursts. An additional 10 s burst was performed after suspending the samples in ICL buffer (10 mM Tris-HCl, pH 8.8, 0.2 M NaCl, 30 mM magnesium chloride, 0.2 M sucrose, and 10 mM 2-mercaptoethanol) containing protease inhibitors aprotinin (2 µg/mL), leupeptin (6 µg/mL), chymostatin (6 µg/mL), E64 (6 µg/mL), 4-(2-aminoethyl)benzenesulfonyl fluoride (25 µg/mL), and 2 mM EDTA. Water-insoluble material was pelleted by centrifugation for 10 min at 10,000× *g* at 4 °C, and the supernatant obtained represented the water-soluble fraction of the intracellular proteins. Pellets obtained in the previous step (the membrane protein fraction) were solubilized in Sample Buffer and boiled for 10 min prior to analysis by PAGE.

### 4.4. In Planta Proxomity-Dependent Biotinylation Assay and Isolation of Biotinylated Proteins

Leaf pieces (approximately 30 mg each) from *N. benthamiana* plants transiently producing either *Nt*Phyt-TurboID or SP-TurboID were prepared 2–3 d post-agroinfiltration. Leaf pieces were vacuum infiltrated with buffer containing 50 mM Tris-HCl, pH 7.5, 1.25 mM magnesium acetate, 0.5 mM ATP, and 200 µM biotin and incubated in the dark at room temperature for 4 h. The apoplastic liquid was then removed by centrifugation at 2000× *g* for 10 min at 4 °C, and leaf pieces were frozen in liquid nitrogen and homogenized. The homogenates were suspended in ICL buffer containing protease inhibitors and incubated on ice for 15 min. Water-insoluble material was pelleted by centrifugation for 10 min at 10,000× *g* at 4 °C and re-suspended in ICL buffer containing 0.5% DDM and 1% SDS. After incubation for 15 min on ice, the supernatant representing the membrane protein fraction was obtained by centrifugation for 10 min at 10,000× *g* at 4 °C.

The detergent-soluble proteins were further fractionated by ammonium sulfate precipitation. The protein fraction that precipitated within the 0–50% interval of (NH_4_)_2_SO_4_ saturation was collected by centrifugation for 20 min at 12,000× *g* at 4 °C and dissolved in 50 mM Tris-HCl buffer (pH 7.5) containing 0.5% DDM, 1% SDS, and protease inhibitors. Biotinylated proteins from these samples were then separated by affinity chromatography using Streptavidin Magnetic Beads (New England Biolabs, Ipswich, MA, USA). The protein samples were boiled for 5 min and diluted two-fold with wash buffer (50 mM Tris-HCl, pH 7.5, 0.05% Tween 20, 0.5 M NaCl) prior to their addition to magnetic beads (200 μL of an aqueous suspension per experiment, pre-washed with Wash buffer). After incubation for 4 h at room temperature with rotation, beads with bound biotinylated proteins were separated with a magnet, washed with Wash buffer supplemented with 4 M urea and 1 mM DTT (4 × 800 μL), and biotinylated proteins were eluted from the beads with 100 μL of elution mix (50 mM Tris-HCl, pH 8.5, 5% SDS) by incubating the samples at 100 °C for 7 min.

The eluates were applied in aliquots to S-Trap Micro Spin columns (Protifi, Fairport, NY, USA) followed by centrifugation at 4000× *g* for 30 s. The columns were then washed with 4 × 125 μL binding buffer (100 mM Tris-HCl, pH 7.5, 90% methanol) and, after the addition of trypsin (1 μg in 20 μL of 50 mM NH_4_HCO_3_), incubated at 37 °C for 16 h. Tryptic peptides were sequentially eluted from the columns with 50 mM NH_4_HCO_3_, 0.2% formic acid, and 50% CH_3_CN (40 μL each, followed by centrifugation at 4000× *g* for 1 min). The combined eluates were vacuum-dried and dissolved in 0.1% formic acid.

### 4.5. LC-MS Analysis

One microgram of peptides in a volume of 1–4 µL was loaded onto the Acclaim µ-Precolumn (0.5 mm × 3 mm, 5 µm particle size, Thermo Scientific, Rockwell, IL, USA) at a flow rate of 10 µL/min for 4 min in an isocratic mode of mobile phase C (2% acetonitrile, 0.1% formic acid). Then, the peptides were separated by high-performance liquid chromatography (HPLC, Ultimate 3000 Nano LC System, Thermo Scientific, Rockwell, IL, USA) in a 20 cm long C18 column (Peaky, inner diameter of 100 μm, Molecta, Moscow, Russia). The peptides were eluted with a gradient of buffer B (80% acetonitrile, 0.1% formic acid) at a flow rate of 0.3 μL/min. The total run time was 90 min, which included an initial 4 min of column equilibration with buffer A (0.1% formic acid), a gradient from 5 to 40% of buffer B over 65 min, then 6 min to reach 99% of buffer B, 10 min of flushing with 99% of buffer B, and 5 min of re-equilibration with buffer A.

MS analysis was performed at least in triplicate with a Q Exactive HFX mass spectrometer (Q Exactive HFX Hybrid Quadrupole-OrbitrapTM mass spectrometer, Thermo Fisher Scientific, Rockwell, IL, USA). The temperature of the capillary was 240 °C, and the voltage at the emitter was 2.1 kV. Mass spectra were acquired at a resolution of 120,000 (MS) in a range of 390–1500 *m*/*z*. Tandem mass spectra of fragments were acquired at a resolution of 60,000 (MS/MS) in the range from 120 *m*/*z* to an *m*/*z* value determined by the charge state of the precursor. The maximum integration times were 50 ms and 110 ms for precursor and fragment ions, respectively. The AGC targets for precursor and fragment ions were set to 1 × 10^6^ and 1 × 10^5^, respectively. An isolation intensity threshold of 400,000 counts was determined for precursor selection, and up to the top 20 precursors were chosen for fragmentation with high-energy collisional dissociation (HCD) at 29 NCE. Precursors with a charge state of +1 and greater than +5 were rejected, and all measured precursors were dynamically excluded from triggering subsequent MS/MS for 40 s.

The MaxQuant search engine (v.2.0.3.0) was used to analyze the resultant data. Proteins were identified by the built-in Andromeda algorithm using the *N. benthamiana* NbDE dataset, 2019 [49], concatenated with a reverse decoy database. Trypsin was specified as the cleavage enzyme, allowing up to two missed cleavages. The mass tolerances for precursor ions were set to 20 ppm in first search and 5 ppm in the main search, respectively. Carbamidomethyl of Cys was specified as a fixed modification, and oxidation of Met was specified as a variable modification. The “Match between runs” option was also applied.

### 4.6. Production of Recombinant Proteins in Escherichia coli Cells and In Vitro Binding Assay

*E. coli* BL21 (DE3) cells were transformed with pET28a-based constructs encoding either MBP_N-CAS, MBP_C-CAS, or free MBP and grown in 40 mL of LB medium containing 0.2% glucose and 25 μg/mL kanamycin at 37 °C with shaking. Exponentially growing cells were shifted to 18 °C, induced with 0.5 mM isopropyl-β-D-thiogalactoside, and further incubated overnight at 18 °C with shaking. Cells were pelleted by centrifugation at 4000× *g* for 15 min at 4 °C, resuspended in 5 mL of CB buffer (20 mM Tris-HCl, pH 7.4, 200 mM NaCl, 1 mM DTT, 1 mM EDTA) containing the protease inhibitor cocktail cOmplete (Roche Diagnostics GmbH, Mannheim, Germany), sonicated on ice with three 20 s bursts, and centrifuged at 14,000× *g* for 10 min at 4 °C. The cleared lysate (1.0 mL) was incubated with amylose resin (New England Biolabs, Ipswich, MA, USA) (100 μL of an aqueous suspension per experiment, pre-washed with CB buffer) for 1 h at 4 °C with rotation. The resin was then washed with 3 × 1 mL of CB buffer and stored on ice.

For in vitro binding assays, *Nt*Phyt-His was affinity-purified from the apoplast of *Nt*Phyt-overproducing *N. benthamiana* leaves using Ni-NTA agarose affinity chromatography, as described in [37]. To assess CAS-*Nt*Phyt binding, an aliquot (10 μL) of amylose resin with immobilized MBP_N-CAS or MBP_C-CAS (approx. 1 μg protein per binding experiment), or an equivalent amount of the control resin (free MBP), was washed with 3 × 0.5 mL of binding buffer (20 mM MES, pH 5.5, 0.05% Tween 20) and incubated with *Nt*Phyt (approx. 200 ng) in a total volume of 20 μL of the same buffer for 1 h at 4 °C with shaking. Where indicated, *Nt*Phyt was pre-incubated with 100 μM of Ac-VEID-CHO or Ac-DEVD-CHO (from stock solutions in DMSO) or with an equivalent amount of DMSO (mock control) at 25 °C for 30 min prior to its addition to the resin. After centrifugation at 4000× *g* for 90 s, unbound *Nt*Phyt was eliminated by 3 × 200 μL washes with binding buffer, and the resin-bound proteins were eluted by 1 h incubation at 4 °C with 20 μL of CB buffer containing 10 mM maltose. Equal aliquots of the combined eluates were used to determine *Nt*Phyt activity (see below). In parallel, aliquots of eluates were analyzed by SDS gel electrophoresis with Coomassie staining to evaluate the amount of MBP and MBP-CAS fusions. Experiments with *Nt*Phyt binding were performed in triplicate. Data for the *Nt*Phyt proteolytic activity are presented as means ± SD from these experiments.

### 4.7. MALDI-MS Analysis

Reduction of cysteine residues with 10 mM dithiothreitol followed by alkylation with 30 mM iodoacetamide was carried out prior to SDS-PAGE protein separation. Pieces of about 5 mm^3^ of the stained protein-containing gel were destained three times with a 20 mM NH_4_HCO_3_, pH 7.5, 40% aqueous acetonitrile solution. Gel pieces were dehydrated with 200 µL of 100% acetonitrile, and rehydrated with 5 µL of the digestion solution (pH 6) containing 15 µg/mL sequencing-grade trypsin or LysC protease (Promega, Madison, WI, USA) in NH_4_HCO_3_, pH 7.5 aqueous solution. Digestion was carried out at 37 °C for 3 h. The resulting peptides were extracted with 5 µL of a 0.5% TFA (trifluoroacetic acid), 50% acetonitrile solution. An aliquot of 1 μL of in-gel digest extract was mixed with 0.5 µL of 2,5-dihydroxybenzoic acid solution (40 mg/mL in 30% acetonitrile, 0.5% TFA). To characterize short peptides released from the immobilized MBP_N-CAS protein by *Nt*Phyt treatment, the amylose resin was pelleted by centrifugation; the resulting supernatant was cleaned up using ZipTip C18 P10 tips (Millipore, Bedford, MA, USA) and mixed with matrix.

MALDI-TOF (matrix-assisted laser desorption ionization–time-of-flight) MS analysis was performed on an UltrafleXtreme MALDI-TOF-TOF mass spectrometer (Bruker Daltonics, Bremen, Germany). The MH+ molecular ions were measured in reflector mode; the accuracy of monoisotopic mass peak measurement was within 50 ppm. Selected peptides were sequenced by MS/MS using the LIFT mode of the mass spectrometer. Protein identification was carried out by MS ion search with the use of Mascot software version 2.3.02 (Matrix Science, London, UK) against a local protein database. One missed cleavage, Met oxidation, Cys-propionamide or Cys-carbamidomethyl were permitted. Protein scores greater than 64 were considered significant (*p* < 0.05).

### 4.8. Confocal Fluorescence Microscopy

Microscopic visualizations were performed using Nikon C2plus (Nikon, Tokyo, Japan) and Zeiss LSM900 Airyscan (Carl Zeiss, Oberkochen, Germany) confocal microscopes equipped with 486 and 562 nm and 488 and 590 nm laser pairs for excitation of EGFP and mRFP, respectively. For chloroplast autofluorescence detection, a 403 nm laser was used. Images were acquired using CFI Plan Fluor 10×/0.3 and Plan-Apo VC 60×/1.2 water-immersion lenses (Nikon, Tokyo, Japan) and Plan-Apochromat 20×/0.8 and Plan-Apochromat 63×/1.40 Oil DIC oil-immersion lenses (Carl Zeiss, Oberkochen, Germany). Confocal images were processed in NIS Elements Viewer (Nikon, Tokyo, Japan) and ZEN Blue v. 3.4.91 (Carl Zeiss, Oberkochen, Germany), respectively, and arranged and annotated using the GNU Image Manipulation Program (GIMP), version 3.2.4 Community, Free Software (GPLv3), 2026 (https://www.gimp.org, accessed on 15 September 2026).

### 4.9. Phytaspase Activity Determination

Proteolytic activity of phytaspase in total *N. benthamiana* leaf extracts, in chloroplast lysates, and in eluates from the affinity resin was determined using the Ac-VEID-AFC [AFC, 7-amino-4-(trifluoromethyl) coumarin] fluorogenic peptide substrate (California Peptide Research, Salt Lake City, UT, USA) as described in [50]. The peptide substrate was used at a final concentration of 40 μM. The FLUOstar OPTIMA reader (BMG Labtech, Ortenberg, Germany) equipped with 405 nm excitation and 520 nm emission filters was used to quantitate fluorescence intensities. Data are presented as means ± SD from three independent experiments.

### 4.10. Virus-Induced Gene Silencing

*Agrobacterium* C58C1 cultures containing TRV1 and either TRV2:NbCAS_UTR or TRV2:NbCAS_ORF were mixed at a 1:1 ratio and infiltrated into *N. benthamiana* leaves of 2- to 3-week-old plants. TRV1 with an empty TRV2 vector (TRV:00) served as a control. Typically, one month after starting viral infection, total RNA samples were obtained from both types of plants using the RNeasy Plant Mini Kit (Qiagen, Hilden, Germany), reverse transcribed using Magnus reverse transcriptase (Evrogen, Moscow, Russia), and used to determine relative levels of NbCAS mRNA. Real-time qPCR reactions were performed with qPCRmix-HS SYBR (Evrogen, Moscow, Russia) and NbCAS3_qPCR2_dir and NbCAS3_qPCR2_rev primers using the C1000 Thermal Cycler equipped with the CFX96 Real-Time System (Bio-Rad Laboratories, Hercules, CA, USA). The 2^−∆∆Ct^ method was used to calculate the relative changes in gene expression using 18S rRNA as a reference. Typically, a five- to ten-fold reduction in the CAS mRNA level was achieved.

Leaves of the silenced and control plants were then infiltrated with INF1 protein (100 nM) [51,52] and monitored over the next few days. Alternatively, leaves were infiltrated with agrobacteria bearing the protein-encoding (*Nt*Phyt-mRFP) construct. At 2 dpi, leaves were treated with 50 µM antimycin A overnight in the dark and inspected using confocal fluorescence microscopy.

### 4.11. Western Blot Analysis

After the addition of 5× sample buffer (250 mM Tris-HCl, pH 6.8, 5 mM EDTA, 10% SDS, 30% glycerol, 50 mM DTT), samples were boiled for 10 min and fractionated by SDS-polyacrylamide gel electrophoresis using 10% gels [53]. Separated proteins were electrophoretically transferred onto polyvinylidene fluoride (PVDF) membranes. Detection of the EGFP-containing proteins was performed using the monoclonal anti-EGFP 3A9 antibody (at a 1:2000 dilution) as described previously [14]. Chemiluminescence detection was performed with Clarity Western ECL Substrate using the ChemiDoc Imaging System (Bio-Rad Laboratories, Hercules, CA, USA).

## Data Availability

Data are contained within the article and Supplementary Materials.

## Supplementary Materials

The following supporting information can be downloaded at: https://www.mdpi.com/article/10.3390/ijms27209202/s1.
