# Supplementary material for: Identification of Calcium-Sensing Receptor (CAS) as a Phytaspase Interactor: Implications for the CAS Trafficking Pathway
Source: Int J Mol Sci. 2026 Oct 16;27(20):9202. doi: 10.3390/ijms27209202 (PMC13705120; doi:10.3390/ijms27209202)
Supplement: Supplementary file 1 [file ijms-27-09202-s001.zip › ijms-4593847-supplementary.pdf]

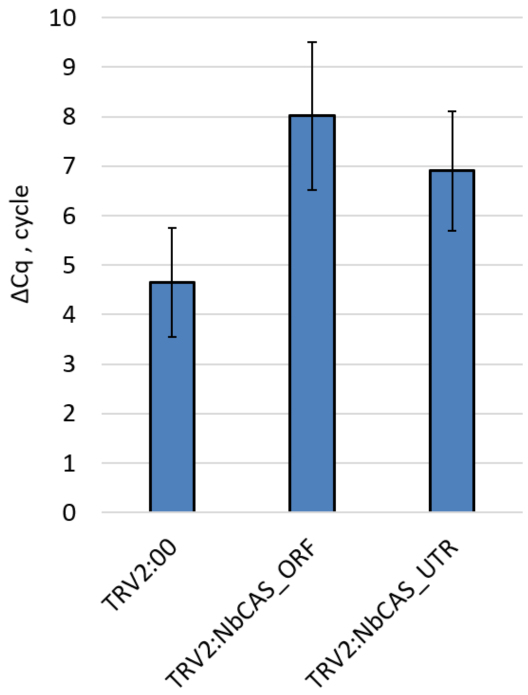

Supplementary Figure S1. Down-regulation of *Nb*CAS mRNA levels in *N. benthamiana* leaves achieved with TRV2:NbCAS_ORF and TRV2:NbCAS_UTR constructs, relative to the empty TRV2 vector (TRV2:00). Relative changes in gene expression were calculated using 18S rRNA as a reference. Data represent the mean ± SD. The differences between TRV2:00 (N=13) and TRV2: NbCAS_ORF (N=5) or TRV2:NbCAS_UTR (N=8) were statistically significant (p<0,05). The Wilcoxon rank-sum test was applied with Benjamini-Hochberg correction.

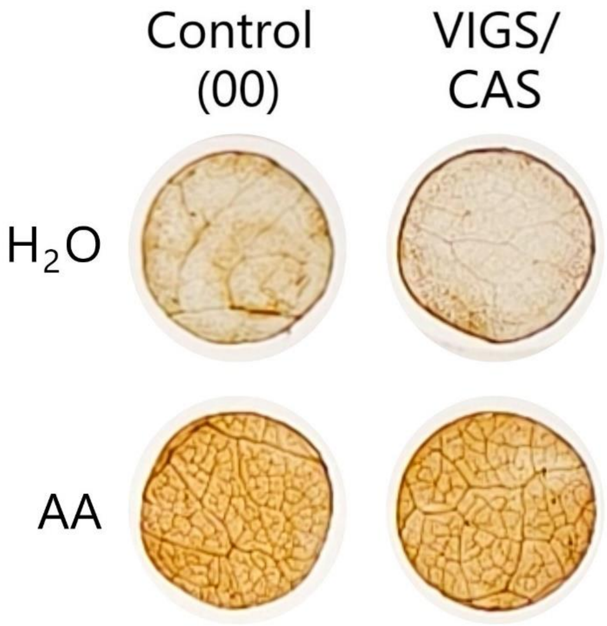

Supplementary Figure S2. The antimycin A-induced generation of reactive oxygen species (ROS) is not impaired in the CAS-silenced plants For ROS detection, leaves of the CAS-silenced (VIGS/CAS) and control (00) *N. benthamiana* plants were treated with 50 µM antimycin A or mock-treated and incubated in the dark for 12 h. Leaf pieces were then vacuum infiltrated with 1 mg/mL DAB (3,3’-diaminobenzidine; Sigma, St. Louis, MO, USA) in 10 mM Na_2HPO_4 and incubated in DAB solution with gentle agitation for 12 h in the dark. The samples w then bleached by incubating in 96% ethanol for 20

min with heating. Ethanol was removed by washing with 70, 50, and 20% ethanol and water, successively. Photographs were taken with bottom illumination.

Supplementary Table S1. Tryptic peptides of *Nb*CAS identified using a LC-MS approach.

	Sequence	Start position	End position	*m/z*
**1**	EDIVSSLNQVESAVNQAQEVGSNIFDAASR	70	99	3176,52
**2**	VIGPVIEFVKPGIDVALPLVK	100	120	2202,34
**3**	NASPVISDATK	129	139	1101,57
**4**	NASPVISDATKK	129	140	1229,66
**5**	KAQEAMQSAGMDAQPVMTAAK	140	160	2163,01
**6**	AQEAMQSAGMDAQPVMTAAK	141	160	2034,92
**7**	TVVDAAQQTSK	161	171	1146,59
**8**	NYVLIDIR	240	247	1004,57
**9**	MIQIPLEDLPSK	268	279	1382,75
**10**	MIQIPLEDLPSKLR	268	281	1651,93
**11**	KVEAELVALK	289	298	1098,66
**12**	VEAELVALK	290	298	970,57
**13**	GSNIVIMDSYSDSAK	308	322	1585,73
**14**	TLTSLGFK	327	334	865,491
**15**	LGTDSYNFSFAQVLSPSR	354	371	1987,96

Supplementary Table S2. The phytaspase cleavage site motifs are relatively well conserved in the CAS proteins of various plant species.

Spinacia oleracea	S V F D	P G I D	L I S E	T V T S	L G T E
Ananas comosus	K V V D	P G V D	V V S D	T V A D	L G T D
Nicotiana tabacum	N I F D	P G V D	V I S D	T V V D	L G T D
Solanum lycopersicum	N I F D	P G I D	V I S D	T V V D	L G T D
Solanum tuberosum	N I F D	P G I D	V I S D	T V V D	L G T D
Papaver somniferum	G V F D	P T V D	V F S E	T V A G	L G T D
Punica granatum	G F L D	P A - -	A I S E	T V G A	L G T D
Daucus carota	N V F D	P G V D	A I S E	T V A D	L G T D
Arabidopsis thaliana	S V F D	P A L D	A F S E	T V T D	L G T D
Vitis vinifera	G F L D	P G I D	A I S E	T V T E	L G T D
Salix viminalis	S V L E	P G I D	A I S E	K V V G	L G A D
Acer saccharum	S F F D	P A I D	A I S E	T V A D	L G A D
Arachis hypogaea	S F L D	P G I D	A I S E	T V V D	L G T D
Glycine soja	G V L D	P G I E	A I S E	T V A D	L G T D
Quercus suber	S F F D	P G I E	A I S E	T V A D	L G T D
Prunus avium	T V F E	P G I D	T I S E	T V A D	L G A D
Malus domestica	T V F D	P G I D	A I S E	T V A D	L G A D
	**NIFD'99**	**PGVD'117**	**VISD'140**	**TVVD'168**	**LGTD'362**

Supplementary Table S3.

List of the primers used in this study.

#	Primer name	Sequence
1	CAS_Kpn_Nco_dir	GAC CCA TGG CGC TTA GAG CTT CAG
2	CAS_Bam_rev	GAA GGA TCC CCA TCA CTA TCC CCT GAA AGC
3	CAS_Head_Bam_dir	CAC GGA TCC GCG CTT AGA GCT TCA GCC AC
4	CAS_Head_Sac_rev	GTC GAG CTC TTA TGG ATC AGC TGA TGA AAT TG
5	CAS_Tail_Bam_dir	CAC GGA TCC AGC CTT CGT GGT TAC AAG G
6	CAS_Tail_Sac_rev	CAC GAG CTC TTA ATC ACT ATC CCC TGA AAG C
7	CAS2_D99E_dir	GGT TCG AAC ATC TTT GAA GCT GCA AGC CGA G
8	CAS2_D117E_rev	GGC AAT GCC ACT TCA ACT CCG GGC TTC
9	CAS2_D140E_dir	GCT TCT CCT GTG ATA TCT GAA GCC ACT AAG AAA GCC C
10	CAS2_D168E_dir	GAC AGT TGT TGA AGC AGC TCA ACA G
11	NbCAS3_ORFVIGS_Kpn_dir	GCA GGT ACC ATG GAC GCT CAA CCA GTG ATG
12	NbCAS3_ORFVIGS_Bam_rev	CAG GGA TCC CTC TGT TCT AAT ATC AAT CAA GAC ATA ATT
13	NbCaS3_VIGS_Kpn_dir	CAA GGT ACC AGT CTC CCC CTT GTA ATG ACC
14	NbCaS3_VIGS_Bam_rev	CAA GGA TCC AAT TGC TTT GAC GAT GAC CGC
15	NbCaS3_qPCR2_dir	AGG AGT CTC GTG AGA AAT GCG
16	NbCaS3_qPCR2_rev	ACC CTT TGT TGA TTT TCT TGA GGT
